# The Management of Acute Gonorrhea

**Published:** 1906-06

**Authors:** Louis Broter

**Affiliations:** New York, Former Assistant, Genito-Urinary Department, Good Samitarian Dispensary


### The Management of Acute Gonorrhea.

By Louis Broter, M.D., New York,
Former Assistant, Genito-Urinary Department, Good Samitarian Dispensary.

AMONG all diseases known to medical men there are few, if any, for which so many remedies have been advocated and used as gonorrhea. It would be more than useless for me to describe them here, for in this brief article space is only given for the latest and best methods of treating this disease as derived from the exeprience of well-known men on this subject and my own experience as well.

When a patient comes to a physician's office with a discharge from his urethra, the physician should not express opinion that it is gonorrhea unless microscopical examination is made and gonococci are found, for if it is a non-infectious urethritis the duration, course and prognosis will vary widely from that of a true gonorrhea. The treatment of a non-infectious urethritis will also differ decidedly from that of infectious urethritis. As a rule, the only treatment necessary in non-infectious urethritis is removal of the cause, rest, and an alkali to ensure a bland condition of urine. After the discharge is examined and gonococci are found, treatment should be instituted in the following way:

*Abortive treatment.*—Many abortive methods are advertised in public newspapers by quacks, some of whom specify the duration of the disease as being from five to eight days, but such rapid cures place the health of the innocent patient in jeopardy. I regret the fate of a patient who falls into the hands of these quacks. The physician must remember that checking a discharge does not necessarily mean curing gonorrhea. For a number of years experiments were made with various drugs such as silver nitrate, bichloride of mercury, etc., with the idea of immediately killing the gonococci, but it has been all in vain. They have only succeeded in producing lesions of various kinds in the urethra, setting up all kinds of complications, thus prolonging the duration of the disease. Those cases which were reported as aborted were either non-infectious or pronounced cured when the discharge had ceased. It is now known to every intelligent practitioner that the discharge may disappear and return after an elapse of a few weeks, for the gonococci penetrate the surface of the mucous membrane of the urethra and remain lodged in the deeper tissues; hence attempts to kill the gonococci immediately would mean to completely destroy the mucous membrane. This is manifestly an absurd procedure. Let us, therefore, be convinced that at present no remedy is known which will abort a true gonorrhea, and let us hope that in the future we will have a remedy which will not only abort but prevent the occurrence of this disease.

*Local treatment.*—Of all methods known I find the irrigation method the best. In 1894 not a dozen men in the world were using the irrigation treatment in gonorrhea. Many had attempted and discarded it; owing to defective apparatus and ignorance of technic, unfortunate results were obtained. Among the earliest successful men were Felicke, of Budapest; Jenet, of Paris; E. R. W. Frank, of Berlin, and Swinburne, of New York. Afterward, this method of treatment became known as the 'Valentine method,' and still preserves its name. To Valentine belongs the credit of devising a simple apparatus, developing the technic, rendering rules precise, and of writing a number of articles pointing out the advantages and disadvantages of this method. The irrigator is too familiar to the profession to be described.

*Method of irrigation.*—The method of irrigation is worthy of description, for, although thousands of physicians are using this method, many are using it in different ways. After urinary and microscopical examinations are made, the patient is instructed to drop his trousers to his knees, to roll up the shirt and undershirt, thus exposing the abdomen. A rubber apron having a hole in the middle for the penis, thus avoiding soiling of clothes, is then put on, and the patient stands close to a stand having a basin in the center, which I use for this special purpose. The patient's eyes should be directed to the percolator of the irrigator to thus engage his attention. This is especially good for neurotic patients.

The stand is placed to the left of the irrigator. The percolator is raised as high as three feet. Have a standard solution of potassium permanganate, 1-4,000 in 1 quart of water, in the percolator. This may be increased up to 1-1,000 as the disease advances. The temperature should be at least 100° F., and may be increased up to 112-120° F. as the patient becomes accustomed to it. The physician stands near the irrigator and to the right of patient. Several clean glasses should be placed on a stand for testing urine; one glass should contain a solution of oxalic acid to remove stains caused by permanganate; one glass of strong lysol or bichloride solution to keep the nozzles in, so as to prevent reinfection. Before each irrigation the nozzle should be washed with boiling water. After use, place it in lysol or bichloride. At night wash same with hot soda solution. Unless the above precautions are taken the nozzles should not be used, for it would be criminal negligence to produce reinfection.

Take the penis in the left hand between index, middle and thumb fingers; take the stopcock of the irrigator in the right hand; hold the nozzle under boiling water to wash away all microorganisms and dirt. Allow some of the solution to flow on glans, foreskin and meatus. Now direct the stream into the urethra with gradual increase. Should it be desired to wash out the posterior urethra or the bladder, the nozzle must be brought in close proximity to the meatus, at the same time telling the patient to attempt to urinate. This last act, as will be readily understood, loosens the cut-off muscle and the solution gains an entrance. This is continued until an entire quart of the solution is used. After irrigation I inject into the urethra three drams of a two per cent. solution of protargol through an ordinary urethral syringe. The patient is instructed to keep the meatus closed by pinching it with his fingers and he thus retains the solution for from five to ten minutes, after which he allows it to flow out. Some patients will faint during this act. I would, therefore, suggest that they be placed in a recumbent posture, especially the first time. The number of irrigations will vary with each individual case. I usually make it a rule to give irrigations of permanganate twice daily, followed by protargol, until the discharge has lessened and it has changed from a purulent to a serous character. This usually takes from two to three weeks. After this I give irrigations once daily, followed by injections of zinc or lead or both; sometimes I add bismuth to the above two drugs. I find that zinc and lead have a great tendency to check the slight discharge which is seen in the declining stages of the disease. After the urine is clear and the discharge and gonococci have disappeared, I still give irrigations once a week for another month.

I have found that since I began the irrigation method chordee has been absent, no complications have arisen and the duration has been shortened. It is impossible with a three-dram syringe to do away with the mucus, pus and gonococci which become impacted within the urethra during the intervals of treatment. On the other hand, with the irrigation method we are enabled to give the urethra a thorough mechanical cleansing, flushing it out, at the same time killing the gonococci because of the germicidal effect of the potassium permanganate. I believe that every physician who has experience at all with the irrigation treatment will agree with me that this method is one of the greatest blessings that human thought and observation have furnished to mankind.

*Constitutional treatment.* Alkalies and diuretics are best employed to keep the urine unirritating. Bicarbonate of soda in 1 dram doses twice daily may be used. Potassium acetate in 5 to 20 grain doses, t.i.d., is good. A good pill is methylene blue, grain 1; boracic acid, grains 5; one t.i.d. A rather serious objection to this is the discoloration of urine and feces. This last pill serves a two-fold purpose. It is a diuretic and urinary antiseptic. It should be given only during the first two weeks. After this time I give oleum santali, balsam copaibæ or the various preparations of cubebs, in capsule form.

There is little room for doubt that hygienic environment is of greatest importance. The patient should rest in bed most of his time in order to lessen congestion of the parts. All exercises, such as dancing, bicycling, horseback and running, should be avoided. Avoid sexual excitement entirely. Daily movement of the bowels is important. No beverages of any kind such as beer, whiskey, ginger ale, etc., should be used, for they are genito-urinary irritants. The patient should avoid fats, meats, acids or sweets, or any food difficult of digestion. Smoking is to be avoided. Hot milk is very good. It is the duty of the physician to get out all superstitions and fictions which some of them have learned from their friends, such as: (1) "Drinking away a clap," *i.e.,* drinking all kinds of beverages, thus causing disease to disappear. (2) "Giving away a clap," *i.e.*, having sexual intercourse during disease, thus leaving it with the woman. (3) "Purifying the system,"*i.e.*, allowing the discharge to run for at least one or two weeks, thus purifying the system.

Unfortunately, these patients come under the physician's observation after they have undergone all the above mentioned experiments.—*Medical News.*

